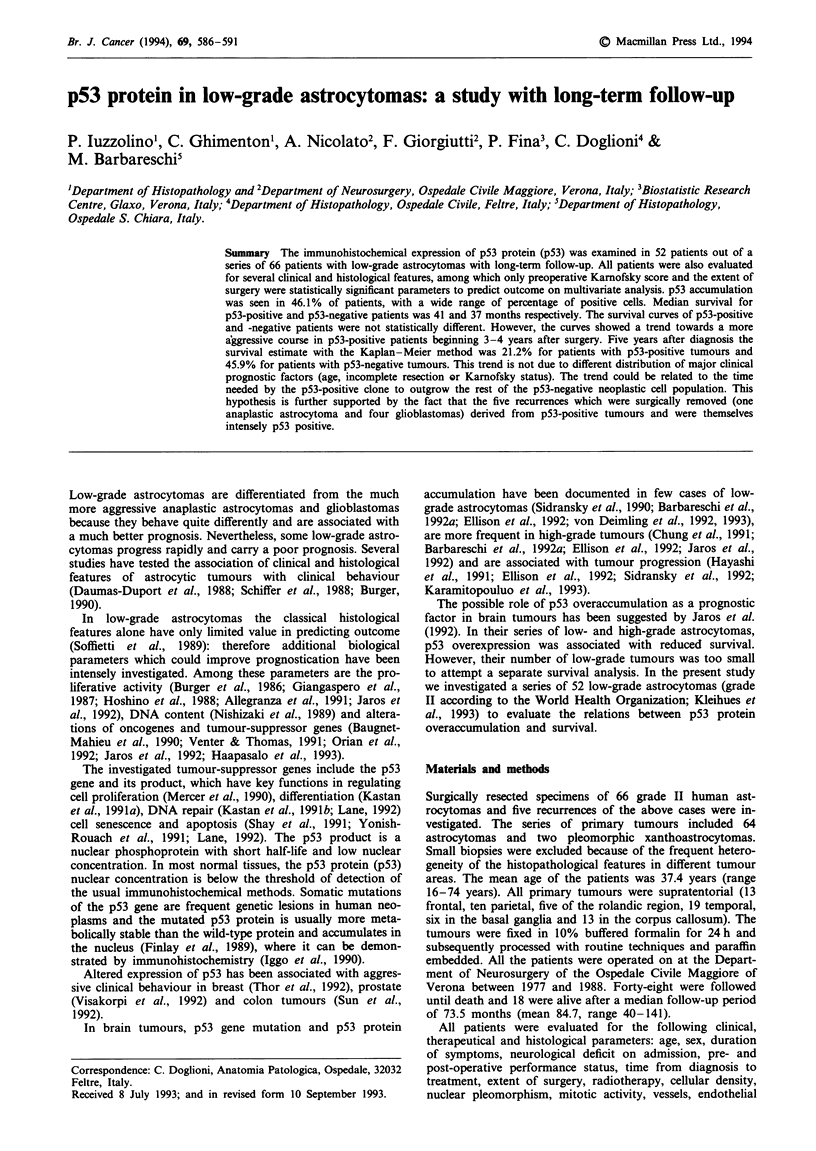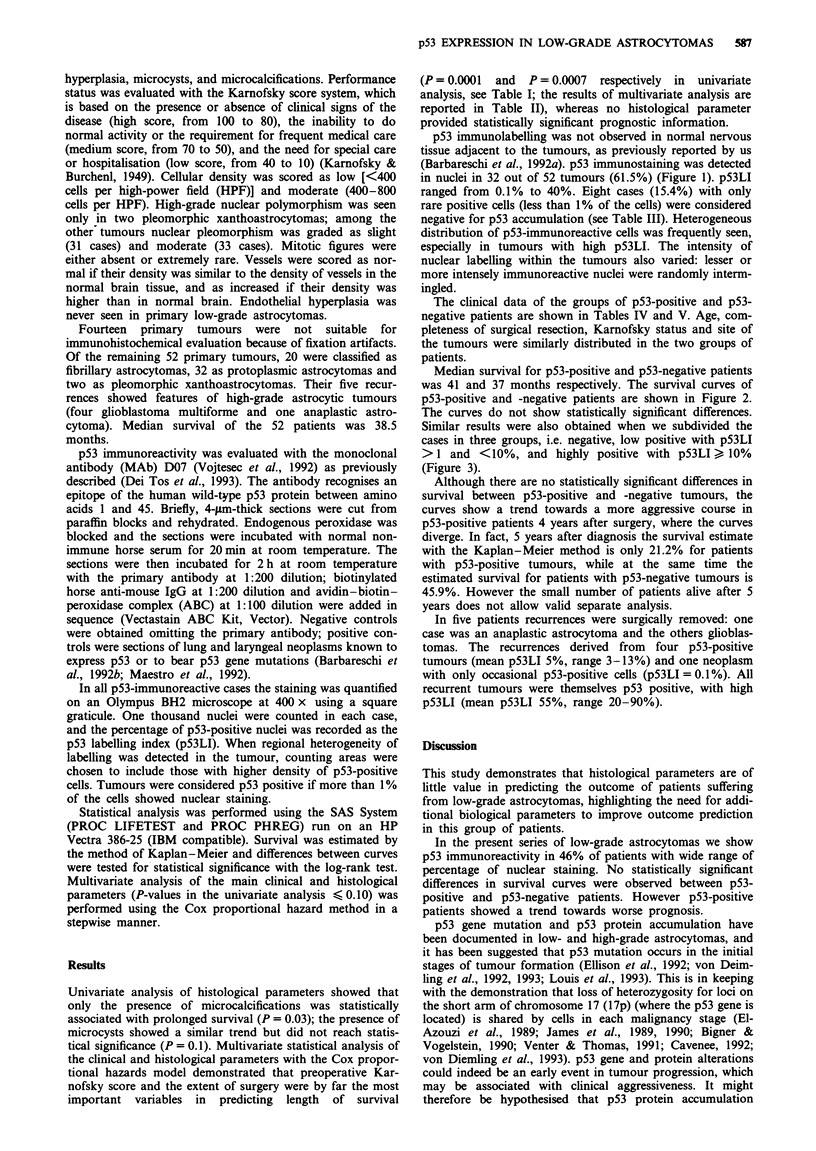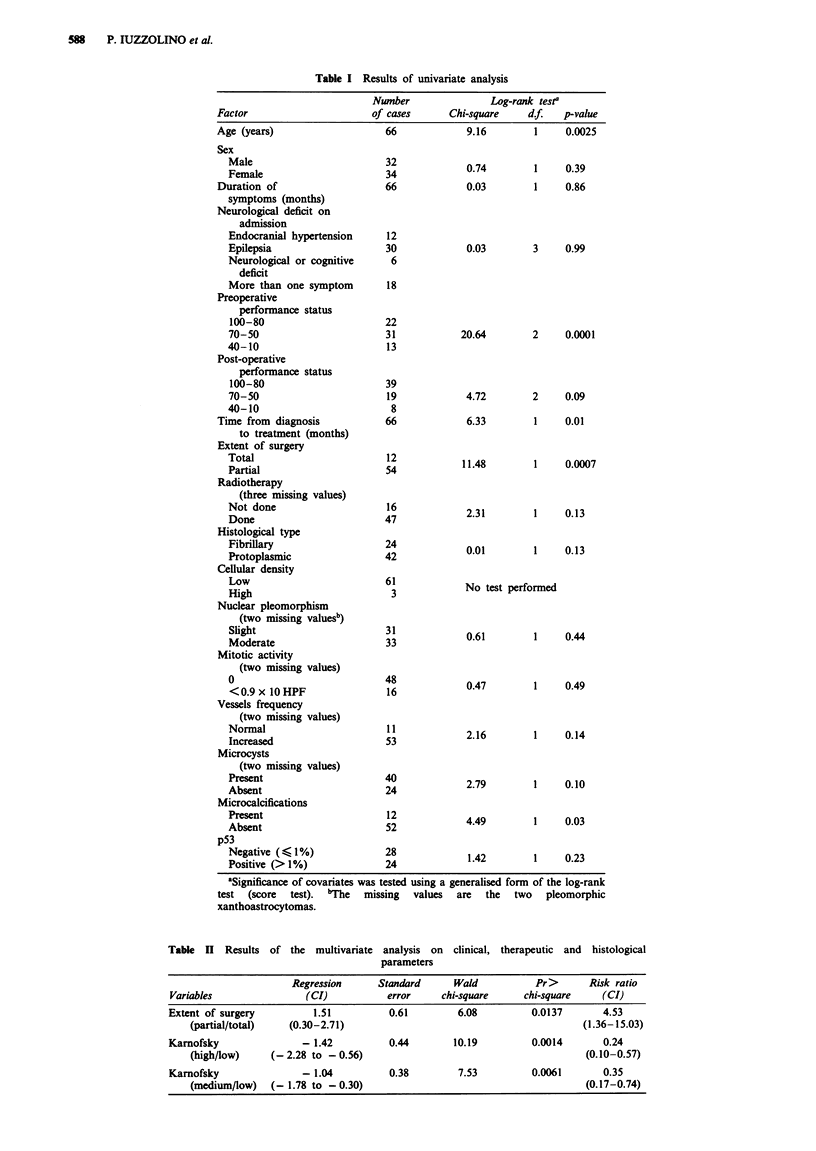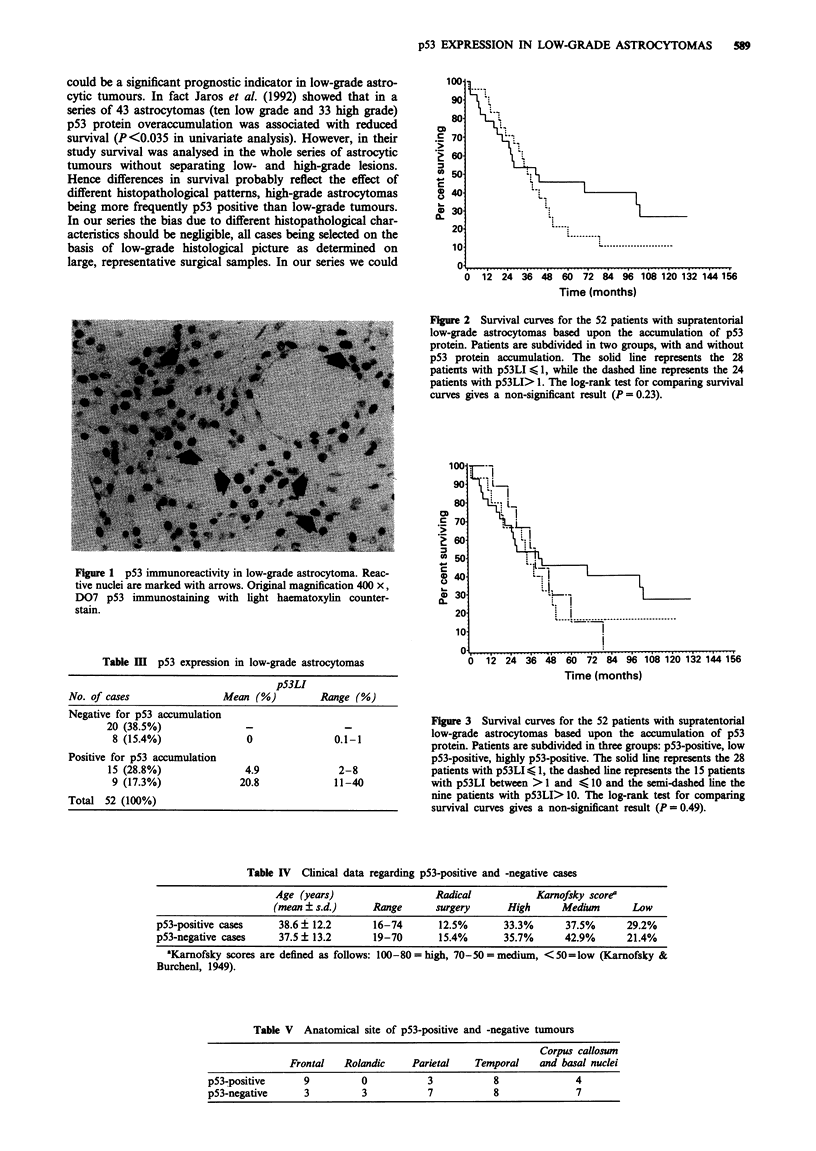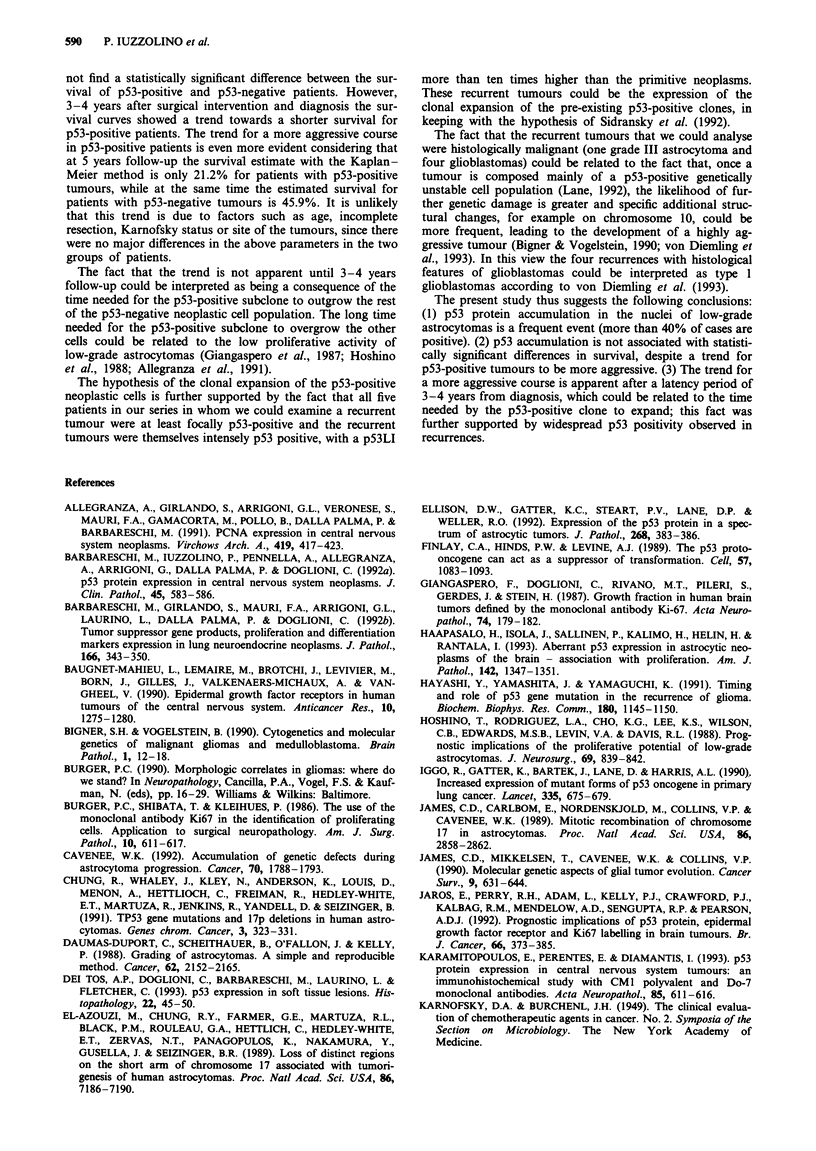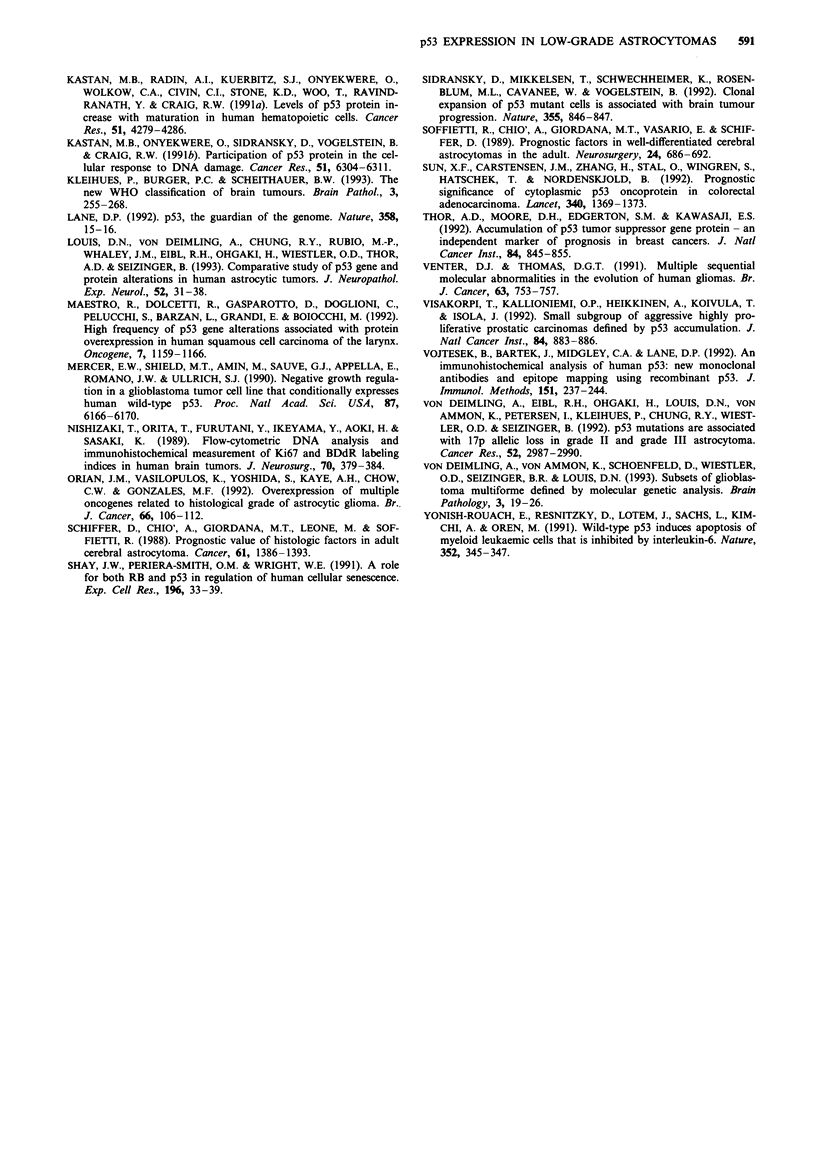# p53 protein in low-grade astrocytomas: a study with long-term follow-up.

**DOI:** 10.1038/bjc.1994.107

**Published:** 1994-03

**Authors:** P. Iuzzolino, C. Ghimenton, A. Nicolato, F. Giorgiutti, P. Fina, C. Doglioni, M. Barbareschi

**Affiliations:** Department of Histopathology, Ospedale Civile Maggiore, Verona, Italy.

## Abstract

**Images:**


Br. J. Cancer (1994), 69, 586-591 © Macmillan Press Ltd., 1994

### p53 protein in low-grade astrocytomas: a study with long-term follow-up

P. Iuzzolino[1], C. Ghimenton[1], A. Nicolato[2], F. Giorgiutti[2], P. Fina[3], C. Doglioni[4] & M. Barbareschi[5]

[1]*Department of Histopathology and* [2]*Department of Neurosurgery, Ospedale Civile Maggiore, Verona, Italy;* [3]*Biostatistic Research Centre, Glaxo, Verona, Italy;* [4]*Department of Histopathology, Ospedale Civile, Feltre, Italy;* [5]*Department of Histopathology, Ospedale S. Chiara, Italy.*

**Summary** The immunohistochemical expression of p53 protein (p53) was examined in 52 patients out of a series of 66 patients with low-grade astrocytomas with long-term follow-up. All patients were also evaluated for several clinical and histological features, among which only preoperative Karnofsky score and the extent of surgery were statistically significant parameters to predict outcome on multivariate analysis. p53 accumulation was seen in 46.1% of patients, with a wide range of percentage of positive cells. Median survival for p53-positive and p53-negative patients was 41 and 37 months respectively. The survival curves of p53-positive and -negative patients were not statistically different. However, the curves showed a trend towards a more aggressive course in p53-positive patients beginning 3-4 years after surgery. Five years after diagnosis the survival estimate with the Kaplan-Meier method was 21.2% for patients with p53-positive tumours and 45.9% for patients with p53-negative tumours. This trend is not due to different distribution of major clinical prognostic factors (age, incomplete resection or Karnofsky status). The trend could be related to the time needed by the p53-positive clone to outgrow the rest of the p53-negative neoplastic cell population. This hypothesis is further supported by the fact that the five recurrences which were surgically removed (one anaplastic astrocytoma and four glioblastomas) derived from p53-positive tumours and were themselves intensely p53 positive.

Low-grade astrocytomas are differentiated from the much more aggressive anaplastic astrocytomas and glioblastomas because they behave quite differently and are associated with a much better prognosis. Nevertheless, some low-grade astrocytomas progress rapidly and carry a poor prognosis. Several studies have tested the association of clinical and histological features of astrocytic tumours with clinical behaviour (Daumas-Duport *et al.*, 1988; Schiffer *et al.*, 1988; Burger, 1990).

In low-grade astrocytomas the classical histological features alone have only limited value in predicting outcome (Soffietti *et al.*, 1989): therefore additional biological parameters which could improve prognostication have been intensely investigated. Among these parameters are the proliferative activity (Burger *et al.*, 1986; Giangaspero *et al.*, 1987; Hoshino *et al.*, 1988; Allegranza *et al.*, 1991; Jaros *et al.*, 1992), DNA content (Nishizaki *et al.*, 1989) and alterations of oncogenes and tumour-suppressor genes (Baugnet-Mahieu *et al.*, 1990; Venter & Thomas, 1991; Orian *et al.*, 1992; Jaros *et al.*, 1992; Haapasalo *et al.*, 1993).

The investigated tumour-suppressor genes include the p53 gene and its product, which have key functions in regulating cell proliferation (Mercer *et al.*, 1990), differentiation (Kastan *et al.*, 1991*a*), DNA repair (Kastan *et al.*, 1991*b*; Lane, 1992) cell senescence and apoptosis (Shay *et al.*, 1991; Yonish-Rouach *et al.*, 1991; Lane, 1992). The p53 product is a nuclear phosphoprotein with short half-life and low nuclear concentration. In most normal tissues, the p53 protein (p53) nuclear concentration is below the threshold of detection of the usual immunohistochemical methods. Somatic mutations of the p53 gene are frequent genetic lesions in human neoplasms and the mutated p53 protein is usually more metabolically stable than the wild-type protein and accumulates in the nucleus (Finlay *et al.*, 1989), where it can be demonstrated by immunohistochemistry (Iggo *et al.*, 1990).

Altered expression of p53 has been associated with aggressive clinical behaviour in breast (Thor *et al.*, 1992), prostate (Visakorpi *et al.*, 1992) and colon tumours (Sun *et al.*, 1992).

In brain tumours, p53 gene mutation and p53 protein accumulation have been documented in few cases of low-grade astrocytomas (Sidransky *et al.*, 1990; Barbareschi *et al.*, 1992*a*; Ellison *et al.*, 1992; von Deimling *et al.*, 1992, 1993), are more frequent in high-grade tumours (Chung *et al.*, 1991; Barbareschi *et al.*, 1992*a*; Ellison *et al.*, 1992; Jaros *et al.*, 1992) and are associated with tumour progression (Hayashi *et al.*, 1991; Ellison *et al.*, 1992; Sidransky *et al.*, 1992; Karamitopouluo *et al.*, 1993).

The possible role of p53 overaccumulation as a prognostic factor in brain tumours has been suggested by Jaros *et al.* (1992). In their series of low- and high-grade astrocytomas, p53 overexpression was associated with reduced survival. However, their number of low-grade tumours was too small to attempt a separate survival analysis. In the present study we investigated a series of 52 low-grade astrocytomas (grade II according to the World Health Organization; Kleihues *et al.*, 1993) to evaluate the relations between p53 protein overaccumulation and survival.

### Materials and methods

Surgically resected specimens of 66 grade II human astrocytomas and five recurrences of the above cases were investigated. The series of primary tumours included 64 astrocytomas and two pleomorphic xanthoastrocytomas. Small biopsies were excluded because of the frequent heterogeneity of the histopathological features in different tumour areas. The mean age of the patients was 37.4 years (range 16-74 years). All primary tumours were supratentorial (13 frontal, ten parietal, five of the rolandic region, 19 temporal, six in the basal ganglia and 13 in the corpus callosum). The tumours were fixed in 10% buffered formalin for 24 h and subsequently processed with routine techniques and paraffin embedded. All the patients were operated on at the Department of Neurosurgery of the Ospedale Civile Maggiore of Verona between 1977 and 1988. Forty-eight were followed until death and 18 were alive after a median follow-up period of 73.5 months (mean 84.7, range 40-141).

All patients were evaluated for the following clinical, therapeutical and histological parameters: age, sex, duration of symptoms, neurological deficit on admission, pre- and post-operative performance status, time from diagnosis to treatment, extent of surgery, radiotherapy, cellular density, nuclear pleomorphism, mitotic activity, vessels, endothelial

Correspondence: C. Doglioni, Anatomia Patologica, Ospedale, 32032 Feltre, Italy.
Received 8 July 1993; and in revised form 10 September 1993.

hyperplasia, microcysts, and microcalcifications. Performance status was evaluated with the Karnofsky score system, which is based on the presence or absence of clinical signs of the disease (high score, from 100 to 80), the inability to do normal activity or the requirement for frequent medical care (medium score, from 70 to 50), and the need for special care or hospitalisation (low score, from 40 to 10) (Karnofsky & Burchenl, 1949). Cellular density was scored as low [<400 cells per high-power field (HPF)] and moderate (400–800 cells per HPF). High-grade nuclear polymorphism was seen only in two pleomorphic xanthoastrocytomas; among the other tumours nuclear pleomorphism was graded as slight (31 cases) and moderate (33 cases). Mitotic figures were either absent or extremely rare. Vessels were scored as normal if their density was similar to the density of vessels in the normal brain tissue, and as increased if their density was higher than in normal brain. Endothelial hyperplasia was never seen in primary low-grade astrocytomas.

Fourteen primary tumours were not suitable for immunohistochemical evaluation because of fixation artifacts. Of the remaining 52 primary tumours, 20 were classified as fibrillary astrocytomas, 32 as protoplasmic astrocytomas and two as pleomorphic xanthoastrocytomas. Their five recurrences showed features of high-grade astrocytic tumours (four glioblastoma multiforme and one anaplastic astrocytoma). Median survival of the 52 patients was 38.5 months.

p53 immunoreactivity was evaluated with the monoclonal antibody (MAb) D07 (Vojtesec *et al.*, 1992) as previously described (Dei Tos *et al.*, 1993). The antibody recognises an epitope of the human wild-type p53 protein between amino acids 1 and 45. Briefly, 4-μm-thick sections were cut from paraffin blocks and rehydrated. Endogenous peroxidase was blocked and the sections were incubated with normal non-immune horse serum for 20 min at room temperature. The sections were then incubated for 2 h at room temperature with the primary antibody at 1:200 dilution; biotinylated horse anti-mouse IgG at 1:200 dilution and avidin–biotin–peroxidase complex (ABC) at 1:100 dilution were added in sequence (Vectastain ABC Kit, Vector). Negative controls were obtained omitting the primary antibody; positive controls were sections of lung and laryngeal neoplasms known to express p53 or to bear p53 gene mutations (Barbareschi *et al.*, 1992*b*; Maestro *et al.*, 1992).

In all p53-immunoreactive cases the staining was quantified on an Olympus BH2 microscope at 400 × using a square graticule. One thousand nuclei were counted in each case, and the percentage of p53-positive nuclei was recorded as the p53 labelling index (p53LI). When regional heterogeneity of labelling was detected in the tumour, counting areas were chosen to include those with higher density of p53-positive cells. Tumours were considered p53 positive if more than 1% of the cells showed nuclear staining.

Statistical analysis was performed using the SAS System (PROC LIFETEST and PROC PHREG) run on an HP Vectra 386-25 (IBM compatible). Survival was estimated by the method of Kaplan–Meier and differences between curves were tested for statistical significance with the log-rank test. Multivariate analysis of the main clinical and histological parameters ($P$-values in the univariate analysis $\leqslant 0.10$) was performed using the Cox proportional hazard method in a stepwise manner.

### Results

Univariate analysis of histological parameters showed that only the presence of microcalcifications was statistically associated with prolonged survival ($P = 0.03$); the presence of microcysts showed a similar trend but did not reach statistical significance ($P = 0.1$). Multivariate statistical analysis of the clinical and histological parameters with the Cox proportional hazards model demonstrated that preoperative Karnofsky score and the extent of surgery were by far the most important variables in predicting length of survival ($P = 0.0001$ and $P = 0.0007$ respectively in univariate analysis, see Table I; the results of multivariate analysis are reported in Table II), whereas no histological parameter provided statistically significant prognostic information.

p53 immunolabelling was not observed in normal nervous tissue adjacent to the tumours, as previously reported by us (Barbareschi *et al.*, 1992*a*). p53 immunostaining was detected in nuclei in 32 out of 52 tumours (61.5%) (Figure 1). p53LI ranged from 0.1% to 40%. Eight cases (15.4%) with only rare positive cells (less than 1% of the cells) were considered negative for p53 accumulation (see Table III). Heterogeneous distribution of p53-immunoreactive cells was frequently seen, especially in tumours with high p53LI. The intensity of nuclear labelling within the tumours also varied: lesser or more intensely immunoreactive nuclei were randomly intermingled.

The clinical data of the groups of p53-positive and p53-negative patients are shown in Tables IV and V. Age, completeness of surgical resection, Karnofsky status and site of the tumours were similarly distributed in the two groups of patients.

Median survival for p53-positive and p53-negative patients was 41 and 37 months respectively. The survival curves of p53-positive and -negative patients are shown in Figure 2. The curves do not show statistically significant differences. Similar results were also obtained when we subdivided the cases in three groups, i.e. negative, low positive with p53LI $>1$ and $<10\%$, and highly positive with p53LI $\geqslant 10\%$ (Figure 3).

Although there are no statistically significant differences in survival between p53-positive and -negative tumours, the curves show a trend towards a more aggressive course in p53-positive patients 4 years after surgery, where the curves diverge. In fact, 5 years after diagnosis the survival estimate with the Kaplan–Meier method is only 21.2% for patients with p53-positive tumours, while at the same time the estimated survival for patients with p53-negative tumours is 45.9%. However the small number of patients alive after 5 years does not allow valid separate analysis.

In five patients recurrences were surgically removed: one case was an anaplastic astrocytoma and the others glioblastomas. The recurrences derived from four p53-positive tumours (mean p53LI 5%, range 3–13%) and one neoplasm with only occasional p53-positive cells (p53LI = 0.1%). All recurrent tumours were themselves p53 positive, with high p53LI (mean p53LI 55%, range 20–90%).

### Discussion

This study demonstrates that histological parameters are of little value in predicting the outcome of patients suffering from low-grade astrocytomas, highlighting the need for additional biological parameters to improve outcome prediction in this group of patients.

In the present series of low-grade astrocytomas we show p53 immunoreactivity in 46% of patients with wide range of percentage of nuclear staining. No statistically significant differences in survival curves were observed between p53-positive and p53-negative patients. However p53-positive patients showed a trend towards worse prognosis.

p53 gene mutation and p53 protein accumulation have been documented in low- and high-grade astrocytomas, and it has been suggested that p53 mutation occurs in the initial stages of tumour formation (Ellison *et al.*, 1992; von Deimling *et al.*, 1992, 1993; Louis *et al.*, 1993). This is in keeping with the demonstration that loss of heterozygosity for loci on the short arm of chromosome 17 (17p) (where the p53 gene is located) is shared by cells in each malignancy stage (El-Azouzi *et al.*, 1989; James *et al.*, 1989, 1990; Bigner & Vogelstein, 1990; Venter & Thomas, 1991; Cavenee, 1992; von Diemling *et al.*, 1993). p53 gene and protein alterations could indeed be an early event in tumour progression, which may be associated with clinical aggressiveness. It might therefore be hypothesised that p53 protein accumulation

**Table I** Results of univariate analysis

| Factor | Number of cases | Log-rank test[a] Chi-square | d.f. | p-value |
|---|---|---|---|---|
| Age (years) | 66 | 9.16 | 1 | 0.0025 |
| Sex | | | | |
| Male | 32 | 0.74 | 1 | 0.39 |
| Female | 34 | | | |
| Duration of symptoms (months) | 66 | 0.03 | 1 | 0.86 |
| Neurological deficit on admission | | | | |
| Endocranial hypertension | 12 | | | |
| Epilepsia | 30 | 0.03 | 3 | 0.99 |
| Neurological or cognitive deficit | 6 | | | |
| More than one symptom | 18 | | | |
| Preoperative performance status | | | | |
| 100–80 | 22 | | | |
| 70–50 | 31 | 20.64 | 2 | 0.0001 |
| 40–10 | 13 | | | |
| Post-operative performance status | | | | |
| 100–80 | 39 | | | |
| 70–50 | 19 | 4.72 | 2 | 0.09 |
| 40–10 | 8 | | | |
| Time from diagnosis to treatment (months) | 66 | 6.33 | 1 | 0.01 |
| Extent of surgery | | | | |
| Total | 12 | 11.48 | 1 | 0.0007 |
| Partial | 54 | | | |
| Radiotherapy (three missing values) | | | | |
| Not done | 16 | 2.31 | 1 | 0.13 |
| Done | 47 | | | |
| Histological type | | | | |
| Fibrillary | 24 | 0.01 | 1 | 0.13 |
| Protoplasmic | 42 | | | |
| Cellular density | | | | |
| Low | 61 | No test performed | | |
| High | 3 | | | |
| Nuclear pleomorphism (two missing values[b]) | | | | |
| Slight | 31 | 0.61 | 1 | 0.44 |
| Moderate | 33 | | | |
| Mitotic activity (two missing values) | | | | |
| 0 | 48 | 0.47 | 1 | 0.49 |
| <0.9 × 10 HPF | 16 | | | |
| Vessels frequency (two missing values) | | | | |
| Normal | 11 | 2.16 | 1 | 0.14 |
| Increased | 53 | | | |
| Microcysts (two missing values) | | | | |
| Present | 40 | 2.79 | 1 | 0.10 |
| Absent | 24 | | | |
| Microcalcifications | | | | |
| Present | 12 | 4.49 | 1 | 0.03 |
| Absent | 52 | | | |
| p53 | | | | |
| Negative (⩽1%) | 28 | 1.42 | 1 | 0.23 |
| Positive (>1%) | 24 | | | |

[a]Significance of covariates was tested using a generalised form of the log-rank test (score test). [b]The missing values are the two pleomorphic xanthoastrocytomas.

**Table II** Results of the multivariate analysis on clinical, therapeutic and histological parameters

| Variables | Regression (CI) | Standard error | Wald chi-square | Pr> chi-square | Risk ratio (CI) |
|---|---|---|---|---|---|
| Extent of surgery (partial/total) | 1.51 (0.30–2.71) | 0.61 | 6.08 | 0.0137 | 4.53 (1.36–15.03) |
| Karnofsky (high/low) | − 1.42 (− 2.28 to − 0.56) | 0.44 | 10.19 | 0.0014 | 0.24 (0.10–0.57) |
| Karnofsky (medium/low) | − 1.04 (− 1.78 to − 0.30) | 0.38 | 7.53 | 0.0061 | 0.35 (0.17–0.74) |

could be a significant prognostic indicator in low-grade astrocytic tumours. In fact Jaros *et al.* (1992) showed that in a series of 43 astrocytomas (ten low grade and 33 high grade) p53 protein overaccumulation was associated with reduced survival ($P<0.035$ in univariate analysis). However, in their study survival was analysed in the whole series of astrocytic tumours without separating low- and high-grade lesions. Hence differences in survival probably reflect the effect of different histopathological patterns, high-grade astrocytomas being more frequently p53 positive than low-grade tumours. In our series the bias due to different histopathological characteristics should be negligible, all cases being selected on the basis of low-grade histological picture as determined on large, representative surgical samples. In our series we could

**Figure 1** p53 immunoreactivity in low-grade astrocytoma. Reactive nuclei are marked with arrows. Original magnification 400 ×, DO7 p53 immunostaining with light haematoxylin counterstain.

**Table III** p53 expression in low-grade astrocytomas

| *No. of cases* | *p53LI* *Mean (%)* | *Range (%)* |
|---|---|---|
| Negative for p53 accumulation | | |
| 20 (38.5%) | – | – |
| 8 (15.4%) | 0 | 0.1–1 |
| Positive for p53 accumulation | | |
| 15 (28.8%) | 4.9 | 2–8 |
| 9 (17.3%) | 20.8 | 11–40 |
| Total 52 (100%) | | |

**Figure 2** Survival curves for the 52 patients with supratentorial low-grade astrocytomas based upon the accumulation of p53 protein. Patients are subdivided in two groups, with and without p53 protein accumulation. The solid line represents the 28 patients with p53LI ⩽ 1, while the dashed line represents the 24 patients with p53LI > 1. The log-rank test for comparing survival curves gives a non-significant result ($P = 0.23$).

**Figure 3** Survival curves for the 52 patients with supratentorial low-grade astrocytomas based upon the accumulation of p53 protein. Patients are subdivided in three groups: p53-positive, low p53-positive, highly p53-positive. The solid line represents the 28 patients with p53LI ⩽ 1, the dashed line represents the 15 patients with p53LI between > 1 and ⩽ 10 and the semi-dashed line the nine patients with p53LI > 10. The log-rank test for comparing survival curves gives a non-significant result ($P = 0.49$).

**Table IV** Clinical data regarding p53-positive and -negative cases

| | *Age (years) (mean ± s.d.)* | *Range* | *Radical surgery* | *Karnofsky score*[a] *High* | *Medium* | *Low* |
|---|---|---|---|---|---|---|
| p53-positive cases | 38.6 ± 12.2 | 16–74 | 12.5% | 33.3% | 37.5% | 29.2% |
| p53-negative cases | 37.5 ± 13.2 | 19–70 | 15.4% | 35.7% | 42.9% | 21.4% |

[a]Karnofsky scores are defined as follows: 100–80 = high, 70–50 = medium, < 50 = low (Karnofsky & Burchenl, 1949).

**Table V** Anatomical site of p53-positive and -negative tumours

| | *Frontal* | *Rolandic* | *Parietal* | *Temporal* | *Corpus callosum and basal nuclei* |
|---|---|---|---|---|---|
| p53-positive | 9 | 0 | 3 | 8 | 4 |
| p53-negative | 3 | 3 | 7 | 8 | 7 |

not find a statistically significant difference between the survival of p53-positive and p53-negative patients. However, 3–4 years after surgical intervention and diagnosis the survival curves showed a trend towards a shorter survival for p53-positive patients. The trend for a more aggressive course in p53-positive patients is even more evident considering that at 5 years follow-up the survival estimate with the Kaplan–Meier method is only 21.2% for patients with p53-positive tumours, while at the same time the estimated survival for patients with p53-negative tumours is 45.9%. It is unlikely that this trend is due to factors such as age, incomplete resection, Karnofsky status or site of the tumours, since there were no major differences in the above parameters in the two groups of patients.

The fact that the trend is not apparent until 3–4 years follow-up could be interpreted as being a consequence of the time needed for the p53-positive subclone to outgrow the rest of the p53-negative neoplastic cell population. The long time needed for the p53-positive subclone to overgrow the other cells could be related to the low proliferative activity of low-grade astrocytomas (Giangaspero *et al.*, 1987; Hoshino *et al.*, 1988; Allegranza *et al.*, 1991).

The hypothesis of the clonal expansion of the p53-positive neoplastic cells is further supported by the fact that all five patients in our series in whom we could examine a recurrent tumour were at least focally p53-positive and the recurrent tumours were themselves intensely p53 positive, with a p53LI more than ten times higher than the primitive neoplasms. These recurrent tumours could be the expression of the clonal expansion of the pre-existing p53-positive clones, in keeping with the hypothesis of Sidransky *et al.* (1992).

The fact that the recurrent tumours that we could analyse were histologically malignant (one grade III astrocytoma and four glioblastomas) could be related to the fact that, once a tumour is composed mainly of a p53-positive genetically unstable cell population (Lane, 1992), the likelihood of further genetic damage is greater and specific additional structural changes, for example on chromosome 10, could be more frequent, leading to the development of a highly aggressive tumour (Bigner & Vogelstein, 1990; von Diemling *et al.*, 1993). In this view the four recurrences with histological features of glioblastomas could be interpreted as type 1 glioblastomas according to von Diemling *et al.* (1993).

The present study thus suggests the following conclusions: (1) p53 protein accumulation in the nuclei of low-grade astrocytomas is a frequent event (more than 40% of cases are positive). (2) p53 accumulation is not associated with statistically significant differences in survival, despite a trend for p53-positive tumours to be more aggressive. (3) The trend for a more aggressive course is apparent after a latency period of 3–4 years from diagnosis, which could be related to the time needed by the p53-positive clone to expand; this fact was further supported by widespread p53 positivity observed in recurrences.

### References

ALLEGRANZA, A., GIRLANDO, S., ARRIGONI, G.L., VERONESE, S., MAURI, F.A., GAMACORTA, M., POLLO, B., DALLA PALMA, P. & BARBARESCHI, M. (1991). PCNA expression in central nervous system neoplasms. *Virchows Arch. A.*, **419**, 417–423.

BARBARESCHI, M., IUZZOLINO, P., PENNELLA, A., ALLEGRANZA, A., ARRIGONI, G., DALLA PALMA, P. & DOGLIONI, C. (1992*a*). p53 protein expression in central nervous system neoplasms. *J. Clin. Pathol.*, **45**, 583–586.

BARBARESCHI, M., GIRLANDO, S., MAURI, F.A., ARRIGONI, G.L., LAURINO, L., DALLA PALMA, P. & DOGLIONI, C. (1992*b*). Tumor suppressor gene products, proliferation and differentiation markers expression in lung neuroendocrine neoplasms. *J. Pathol.*, **166**, 343–350.

BAUGNET-MAHIEU, L., LEMAIRE, M., BROTCHI, J., LEVIVIER, M., BORN, J., GILLES, J., VALKENAERS-MICHAUX, A. & VANGHEEL, V. (1990). Epidermal growth factor receptors in human tumours of the central nervous system. *Anticancer Res.*, **10**, 1275–1280.

BIGNER, S.H. & VOGELSTEIN, B. (1990). Cytogenetics and molecular genetics of malignant gliomas and medulloblastoma. *Brain Pathol.*, **1**, 12–18.

BURGER, P.C. (1990). Morphologic correlates in gliomas: where do we stand? In *Neuropathology*, Cancilla, P.A., Vogel, F.S. & Kaufman, N. (eds), pp. 16–29. Williams & Wilkins: Baltimore.

BURGER, P.C., SHIBATA, T. & KLEIHUES, P. (1986). The use of the monoclonal antibody Ki67 in the identification of proliferating cells. Application to surgical neuropathology. *Am. J. Surg. Pathol.*, **10**, 611–617.

CAVENEE, W.K. (1992). Accumulation of genetic defects during astrocytoma progression. *Cancer*, **70**, 1788–1793.

CHUNG, R., WHALEY, J., KLEY, N., ANDERSON, K., LOUIS, D., MENON, A., HETTLIOCH, C., FREIMAN, R., HEDLEY-WHITE, E.T., MARTUZA, R., JENKINS, R., YANDELL, D. & SEIZINGER, B. (1991). TP53 gene mutations and 17p deletions in human astrocytomas. *Genes chrom. Cancer*, **3**, 323–331.

DAUMAS-DUPORT, C., SCHEITHAUER, B., O'FALLON, J. & KELLY, P. (1988). Grading of astrocytomas. A simple and reproducible method. *Cancer*, **62**, 2152–2165.

DEI TOS, A.P., DOGLIONI, C., BARBARESCHI, M., LAURINO, L. & FLETCHER, C. (1993). p53 expression in soft tissue lesions. *Histopathology*, **22**, 45–50.

EL-AZOUZI, M., CHUNG, R.Y., FARMER, G.E., MARTUZA, R.L., BLACK, P.M., ROULEAU, G.A., HETTLICH, C., HEDLEY-WHITE, E.T., ZERVAS, N.T., PANAGOPULOS, K., NAKAMURA, Y., GUSELLA, J. & SEIZINGER, B.R. (1989). Loss of distinct regions on the short arm of chromosome 17 associated with tumorigenesis of human astrocytomas. *Proc. Natl Acad. Sci. USA*, **86**, 7186–7190.

ELLISON, D.W., GATTER, K.C., STEART, P.V., LANE, D.P. & WELLER, R.O. (1992). Expression of the p53 protein in a spectrum of astrocytic tumors. *J. Pathol.*, **268**, 383–386.

FINLAY, C.A., HINDS, P.W. & LEVINE, A.J. (1989). The p53 proto-oncogene can act as a suppressor of transformation. *Cell*, **57**, 1083–1093.

GIANGASPERO, F., DOGLIONI, C., RIVANO, M.T., PILERI, S., GERDES, J. & STEIN, H. (1987). Growth fraction in human brain tumors defined by the monoclonal antibody Ki-67. *Acta Neuropathol.*, **74**, 179–182.

HAAPASALO, H., ISOLA, J., SALLINEN, P., KALIMO, H., HELIN, H. & RANTALA, I. (1993). Aberrant p53 expression in astrocytic neoplasms of the brain – association with proliferation. *Am. J. Pathol.*, **142**, 1347–1351.

HAYASHI, Y., YAMASHITA, J. & YAMAGUCHI, K. (1991). Timing and role of p53 gene mutation in the recurrence of glioma. *Biochem. Biophys. Res. Comm.*, **180**, 1145–1150.

HOSHINO, T., RODRIGUEZ, L.A., CHO, K.G., LEE, K.S., WILSON, C.B., EDWARDS, M.S.B., LEVIN, V.A. & DAVIS, R.L. (1988). Prognostic implications of the proliferative potential of low-grade astrocytomas. *J. Neurosurg.*, **69**, 839–842.

IGGO, R., GATTER, K., BARTEK, J., LANE, D. & HARRIS, A.L. (1990). Increased expression of mutant forms of p53 oncogene in primary lung cancer. *Lancet*, **335**, 675–679.

JAMES, C.D., CARLBOM, E., NORDENSKJOLD, M., COLLINS, V.P. & CAVENEE, W.K. (1989). Mitotic recombination of chromosome 17 in astrocytomas. *Proc. Natl Acad. Sci. USA*, **86**, 2858–2862.

JAMES, C.D., MIKKELSEN, T., CAVENEE, W.K. & COLLINS, V.P. (1990). Molecular genetic aspects of glial tumor evolution. *Cancer Surv.*, **9**, 631–644.

JAROS, E., PERRY, R.H., ADAM, L., KELLY, P.J., CRAWFORD, P.J., KALBAG, R.M., MENDELOW, A.D., SENGUPTA, R.P. & PEARSON, A.D.J. (1992). Prognostic implications of p53 protein, epidermal growth factor receptor and Ki67 labelling in brain tumours. *Br. J. Cancer*, **66**, 373–385.

KARAMITOPOULOS, E., PERENTES, E. & DIAMANTIS, I. (1993). p53 protein expression in central nervous system tumours: an immunohistochemical study with CM1 polyvalent and Do-7 monoclonal antibodies. *Acta Neuropathol.*, **85**, 611–616.

KARNOFSKY, D.A. & BURCHENL, J.H. (1949). The clinical evaluation of chemotherapeutic agents in cancer. No. 2. *Symposia of the Section on Microbiology*. The New York Academy of Medicine.

KASTAN, M.B., RADIN, A.I., KUERBITZ, S.J., ONYEKWERE, O., WOLKOW, C.A., CIVIN, C.I., STONE, K.D., WOO, T., RAVINDRANATH, Y. & CRAIG, R.W. (1991*a*). Levels of p53 protein increase with maturation in human hematopoietic cells. *Cancer Res.*, **51**, 4279-4286.

KASTAN, M.B., ONYEKWERE, O., SIDRANSKY, D., VOGELSTEIN, B. & CRAIG, R.W. (1991*b*). Participation of p53 protein in the cellular response to DNA damage. *Cancer Res.*, **51**, 6304-6311.

KLEIHUES, P., BURGER, P.C. & SCHEITHAUER, B.W. (1993). The new WHO classification of brain tumours. *Brain Pathol.*, **3**, 255-268.

LANE, D.P. (1992). p53, the guardian of the genome. *Nature*, **358**, 15-16.

LOUIS, D.N., VON DEIMLING, A., CHUNG, R.Y., RUBIO, M.-P., WHALEY, J.M., EIBL, R.H., OHGAKI, H., WIESTLER, O.D., THOR, A.D. & SEIZINGER, B. (1993). Comparative study of p53 gene and protein alterations in human astrocytic tumors. *J. Neuropathol. Exp. Neurol.*, **52**, 31-38.

MAESTRO, R., DOLCETTI, R., GASPAROTTO, D., DOGLIONI, C., PELUCCHI, S., BARZAN, L., GRANDI, E. & BOIOCCHI, M. (1992). High frequency of p53 gene alterations associated with protein overexpression in human squamous cell carcinoma of the larynx. *Oncogene*, **7**, 1159-1166.

MERCER, E.W., SHIELD, M.T., AMIN, M., SAUVE, G.J., APPELLA, E., ROMANO, J.W. & ULLRICH, S.J. (1990). Negative growth regulation in a glioblastoma tumor cell line that conditionally expresses human wild-type p53. *Proc. Natl Acad. Sci. USA*, **87**, 6166-6170.

NISHIZAKI, T., ORITA, T., FURUTANI, Y., IKEYAMA, Y., AOKI, H. & SASAKI, K. (1989). Flow-cytometric DNA analysis and immunohistochemical measurement of Ki67 and BDdR labeling indices in human brain tumors. *J. Neurosurg.*, **70**, 379-384.

ORIAN, J.M., VASILOPULOS, K., YOSHIDA, S., KAYE, A.H., CHOW, C.W. & GONZALES, M.F. (1992). Overexpression of multiple oncogenes related to histological grade of astrocytic glioma. *Br. J. Cancer*, **66**, 106-112.

SCHIFFER, D., CHIO', A., GIORDANA, M.T., LEONE, M. & SOFFIETTI, R. (1988). Prognostic value of histologic factors in adult cerebral astrocytoma. *Cancer*, **61**, 1386-1393.

SHAY, J.W., PERIERA-SMITH, O.M. & WRIGHT, W.E. (1991). A role for both RB and p53 in regulation of human cellular senescence. *Exp. Cell Res.*, **196**, 33-39.

SIDRANSKY, D., MIKKELSEN, T., SCHWECHHEIMER, K., ROSENBLUM, M.L., CAVANEE, W. & VOGELSTEIN, B. (1992). Clonal expansion of p53 mutant cells is associated with brain tumour progression. *Nature*, **355**, 846-847.

SOFFIETTI, R., CHIO', A., GIORDANA, M.T., VASARIO, E. & SCHIFFER, D. (1989). Prognostic factors in well-differentiated cerebral astrocytomas in the adult. *Neurosurgery*, **24**, 686-692.

SUN, X.F., CARSTENSEN, J.M., ZHANG, H., STAL, O., WINGREN, S., HATSCHEK, T. & NORDENSKJOLD, B. (1992). Prognostic significance of cytoplasmic p53 oncoprotein in colorectal adenocarcinoma. *Lancet*, **340**, 1369-1373.

THOR, A.D., MOORE, D.H., EDGERTON, S.M. & KAWASAJI, E.S. (1992). Accumulation of p53 tumor suppressor gene protein - an independent marker of prognosis in breast cancers. *J. Natl Cancer Inst.*, **84**, 845-855.

VENTER, D.J. & THOMAS, D.G.T. (1991). Multiple sequential molecular abnormalities in the evolution of human gliomas. *Br. J. Cancer*, **63**, 753-757.

VISAKORPI, T., KALLIONIEMI, O.P., HEIKKINEN, A., KOIVULA, T. & ISOLA, J. (1992). Small subgroup of aggressive highly proliferative prostatic carcinomas defined by p53 accumulation. *J. Natl Cancer Inst.*, **84**, 883-886.

VOJTESEK, B., BARTEK, J., MIDGLEY, C.A. & LANE, D.P. (1992). An immunohistochemical analysis of human p53: new monoclonal antibodies and epitope mapping using recombinant p53. *J. Immunol. Methods*, **151**, 237-244.

VON DEIMLING, A., EIBL, R.H., OHGAKI, H., LOUIS, D.N., VON AMMON, K., PETERSEN, I., KLEIHUES, P., CHUNG, R.Y., WIESTLER, O.D. & SEIZINGER, B. (1992). p53 mutations are associated with 17p allelic loss in grade II and grade III astrocytoma. *Cancer Res.*, **52**, 2987-2990.

VON DEIMLING, A., VON AMMON, K., SCHOENFELD, D., WIESTLER, O.D., SEIZINGER, B.R. & LOUIS, D.N. (1993). Subsets of glioblastoma multiforme defined by molecular genetic analysis. *Brain Pathology*, **3**, 19-26.

YONISH-ROUACH, E., RESNITZKY, D., LOTEM, J., SACHS, L., KIMCHI, A. & OREN, M. (1991). Wild-type p53 induces apoptosis of myeloid leukaemic cells that is inhibited by interleukin-6. *Nature*, **352**, 345-347.